# Multivariate decomposition of gender differentials in successful aging among older adults in India

**DOI:** 10.1186/s12877-023-03753-0

**Published:** 2023-01-31

**Authors:** Shobhit Srivastava, T. Muhammad, Ronak Paul, Kacho Amir Khan

**Affiliations:** International Institute for Population Sciences, Mumbai, Maharashtra India 400088

**Keywords:** Successful aging, Gender, Older adults, India

## Abstract

**Background:**

Rowe and Kahn define successful aging as a high physical, psychological, and social functioning in old age without major diseases. It is considered a viable solution to the burdens placed on healthcare systems and financial and social security in societies with aging population. The present study aimed to determine the prevalence of successful aging and explore the factors contributing to gender differentials in successful aging among older adults in India.

**Methods:**

This study utilized data from the nationally representative Longitudinal Ageing Study in India, conducted in 2017–18. The study is based on a sample of 15,098 older men and 16,366 older women aged 60 years and above. The outcome variable was a dichotomous measure of successful aging with six components including absence of chronic diseases, free from disability, high cognitive ability, free from depressive symptoms, active social engagement in life and free from obesity. Older adults satisfying all these conditions were considered aging successfully. Descriptive and bivariate analyses were carried out. Proportion test was used to evaluate the gender differentials and reflect the statistical significance in the associated factors. Multivariate decomposition analysis was conducted to identify covariates’ contribution in explaining the gender differences in successful aging.

**Results:**

There was a significant gender difference in successful aging among older adults in India (Difference: 8.7%; *p*-value < 0.001] with 34.3% older men and 25.6% older women experiencing successful aging. A proportion of 88% of gender difference in successful aging was explained by the differences in the distribution of characteristics (Coef: 0.082; *p*-value < 0.05). Considerable gender gap in successful aging would be reduced if women had similar levels of work status (28% reduction) to their male counterparts. Bringing the level of frequent physical activity in women to the same levels observed in men would reduce the gender gap by 9%.

**Conclusions:**

The findings suggest that women had a lower score in successful aging, which is attributed to several socioeconomic and behavioural factors including not working status and physical inactivity. More studies must be done to explore the reasons for such differences and what particular factors in low-income countries create differences among older men and women in achieving successful aging.

## Background

The global population aged 65 and above is predicted to rise from 9% in 2020 to 16% by 2025 [1]. India also faces a rapid population aging with a predicted 13% rise in the population aged 60 years and above between 2005 and 2050 [1]. With the rise in the share of the aging population, new concerns emerge, such as increased demand for healthcare facilities, rising medical costs, and a decreasing labour force [2]. To address this in near future, it is important to understand the current status of the aged population in a country which is uniquely different due to its sheer size of the population.

The term “successful aging” was coined in the 1950s and gained popularity in the 1980s [3]. The main characteristics of successful aging are freedom from disease and disability, good cognitive and physical functioning, and social and constructive participation [3, 4]. Although the operationalization was not strictly followed, several studies in developed and developing countries were conducted using the definition of Rowe and Kahn [5]. It was reported that the their definition includes several dimensions of simultaneously assessed health outcomes such as physical, cognitive and social functioning as well as the disease status and thus, a greater input is needed in defining successful aging [6]. Studies on successful aging in Asia have focused on specific components or added new components including nutritional status in Rowe and Kahn’s model and used modified versions [7–9]. Such modifications in Asian studies have limited the comparison of successful aging with developed countries, however may benefit in understanding the degree to which the opportunities of successful aging vary across different subgroups of older population in Asian settings.

Considering health as a major component of successful aging, there are gender variations that can be seen in disability and disease prevalence, degree of physical and mental functioning and health expectancy and mortality [10–12]. Women, on average, outlive men. However, women are more likely than men to rate themselves lower on the physical and psychosocial resources metrics that are frequently used to assess wellbeing in old age [13, 14]. Having a social life, in the form of a sense of belonging, solid social links, and social support is another critical component of aging successfully. The disparity in life expectancy between men and women and women’s proclivity to marry older men has resulted in a global situation where more older men are married while, more senior women are widowed and living alone with more significant disadvantages [15]. This trend of older women living alone is most visible in Western countries, but it is also becoming a norm in developing countries due to young people’s migration to metropolitan areas for jobs, leaving their parents behind [16, 17].

Successful aging is considered a viable solution to the burdens placed on healthcare systems and financial and social security in societies with aging population [18]. Therefore, the present study aims to determine the prevalence of successful aging and explore the factors contributing to gender differentials in successful aging among older adults in India. The study hypothesized significant gender differentials in successful aging among older adults aged 60 years and above in India.

## Material and methods

### Data

This study utilizes data from India’s first nationally representative Longitudinal Ageing Survey in India (LASI) 2017–18, which investigates the health, economics, and social determinants and consequences of population aging in India [19]. The representative sample included 72,250 adults aged 45 and above and their spouses across all states and union territories of India except Sikkim. The Central Ethics Committee on Human Research (CECHR) under the Indian Council of Medical Research (ICMR) extended the necessary guidance, guidelines and ethics approval for conducting the LASI survey.

The LASI adopted a multistage stratified area probability cluster sampling design to select the eventual observation units. Households with at least one member aged 45 and above were taken as the eventual observation unit. The study provides scientific evidence on demographics, household economic status, chronic health conditions, symptom-based health conditions, functional and mental health, biomarkers, health care utilization, work and employment. It enables the cross-state analyses and cross-national analyses of aging, health, economic status and social behaviours and has been designed to evaluate the effect of changing policies and behavioural outcomes in India. Detailed information on the sampling frame is available in the LASI wave-1 Report. The present study is based on a sample of 31,464 older adults (15,098 male and 16,366 female) defined as those aged 60 years and above [19].

### Outcome variable

The outcome variable "successful aging" was dichotomised and was coded as 0 “no” and 1 “yes” [20]. Successful aging differs from region to region, and the present paper defined successful aging based on the composite index created by Rowe and Kahn [20, 21]. The six components were 1. absence of chronic diseases 2. free from disability 3. high cognitive ability 4. free from depressive symptoms 5. active social engagement in life and 6. free from obesity. The older adults satisfying all the above conditions were considered the successful aging group [20]. The six components are as follows:

1. Absence of chronic diseases: Chronic diseases were assessed from the question “Have you been diagnosed with conditions listed below by a doctor?” The illnesses were hypertension, chronic heart diseases, stroke, any chronic lung disease, diabetes, cancer or malignant tumour, any bone/joint disease, any neurological/psychiatric disease or high cholesterol [22]. Respondents were classified as having no chronic diseases if they reported having none of those mentioned above conditions.
2. Free from disability: Activities of Daily Living (ADL) is a term used to refer to normal daily self-care activities (such as movement in bed, changing position from sitting to standing, feeding, bathing, dressing, grooming, personal hygiene). The ability or inability to perform ADLs is used to measure a person’s functional status, especially in the case of people with disabilities and older adults [23, 24]. Respondents were classified as having no disability if they were ADL independent [25].
3. High cognitive ability: Cognitive impairment was measured through five broad domains (memory, orientation, arithmetic function, executive function and object naming). Memory was measured using immediate word recall delayed word recall; orientation was measured using time and place measure; arithmetic function was measured through backward counting, serial seven and computation method; executive function was measured through paper folding and pentagon drawing method, and object naming was lastly done to measure the cognitive impairment among older adults. A composite score of 0–43 was computed using the domain wise measure. The lowest 10th percentile is used as a proxy measure of poor cognitive functioning [19]. The older adults who did not fall into the category of lowest 10th percentile were considered as having high cognitive ability [26].
4. Free from depressive symptoms: The probable major depression among the older adults with symptoms of dysphoria, calculated using the CIDI-SF (Short Form Composite International Diagnostic Interview) score of 3 or more on the scale of 0–10. This scale estimates a probable psychiatric diagnosis of major depression and has been validated in field settings and widely used in population-based health surveys [19, 27]. The score of more than three was categorized as depressed and vice-versa.
5. Active social engagement in life: Social engagement of respondents were assessed based on their participation in the following activities – Eat out of the house (Restaurant/Hotel); Go to park/beach for relaxing/entertainment; Play cards or indoor games; Play outdoor games/sports/exercise/jog/yoga; Visit relatives /friends; Attend cultural performances /shows/Cinema; Attend religious functions /events such as bhajan/satsang/prayer; Attend political/community/organization group meetings; Read books/newspapers/magazines; Watch television/listen to the radio and use a computer for e-mail/net surfing. The respondent was said to be socially engaged if involved in at least one of the activities mentioned above.
6. Free from obesity: Obesity was coded as yes and no. The respondents with a body mass index of 30 and above were categorized as obese [28].

### Explanatory variables

#### Main group variable

Gender was coded as male and female and was considered as the main group variable in the current analysis.

#### Individual factors

Age was coded as young old (60–69 years), old-old (70–79 years), and oldest-old (80 + years). Education was coded as no education/ primary schooling not completed, primary completed, secondary completed, and higher and above. Marital status was coded as currently married, widowed, and others (separated/never married/divorced) [22]. Work status was coded as working, never worked/retired, and currently not working. Living arrangement was coded as living alone, living with a spouse, living with children and living with others. Tobacco and alcohol consumption was coded as no and yes [22]. Physical activity of respondents was assessed based on the question “How often do you take part in sports or vigorous activities, such as running or jogging, swimming, going to a health centre or gym, cycling, or digging with a spade or shovel, heavy lifting, chopping, farm work, fast bicycling, cycling with loads?”. Physical activity status was coded as frequent (every day), rare (more than once a week, once a week, one to three times in a month), and never [22].

#### Household factors

The monthly per capita expenditure (MPCE) quintile was assessed using household consumption data. Sets of 11 and 29 questions on food and non-food items expenses, respectively, were used to canvas the sample households. Food expenditure was collected based on a reference period of seven days, and the non-food cost was compiled based on reference periods of 30 days and 365 days. Food and non-food expenditures have been standardized to the 30-day reference period. The MPCE is computed and used as the summary measure of consumption. The variable was divided into five quintiles, i.e., from poorest to richest [19]. Religion was coded as Hindu, Muslim, Christian, and Others. Caste was recoded as Scheduled Tribe (ST), Scheduled Caste (SC), Other Backward Class (OBC), and others. Caste is a form of social stratification based on the societal position of population groups and is specific to India. The SC and ST group are among India’s most disadvantaged socioeconomic groups and historically belonged to the lowest rung of the now constitutionally abolished Indian caste system. The ST group consists of a predominantly tribal population. The OBC is the group of people who were identified as “educationally, economically and socially backwards” with conditions better than the ST/SC population. The “other” caste category comprises none of the ST, SC, and OBC groups. The place of residence was coded as rural and urban. The region was coded as North, Central, East, Northeast, West, and South.

### Statistical approach

Descriptive analysis along with bivariate analysis was carried out to present the preliminary results. A proportion test was used to evaluate the gender differentials and find the significance level [29]. Further binary logistic regression analysis [30] was used to determine the factors for successful aging among older adults. The results were presented in an adjusted odds ratio (AOR) with 95% confidence interval (CI). In this study, the odds ratio of greater than 1 for a given category of the independent variable denotes higher odds of successful aging given the effect of all other independent variables remain constant.

A multivariate decomposition analysis [31] was used to identify covariates’ contributions, explaining the group differences in average predictions. The decomposition analysis aimed to identify covariates that contributed to the change in successful aging by gender of older adults (male vs female). The multivariate decomposition analysis has two contribution effects: compositional differences (endowments) and the effects of characteristics that differ in the coefficients or behavioural change of responses for the selected predictor variables [32]. The *svyset* command was used in STATA, which controls the analysis for complex survey design and also weights are adjusted, making the results nationally representative.

## Results

### Univariate distribution

Table 1 shows the individual and household characteristics of 15,098 (48%) male and 16,366 (52%) female older adults in India. The mean age for male and female respondents was 69.3 years and 69.1 years, respectively. We observed that nearly 60% of older adults of either gender were in the young-old age group. Further, one in ten, one in two and one in four older men had higher education, never worked/retired and were living with spouses, respectively. A total of 82% of older women had no formal education, 81% either never worked/retired or were currently not working, and 15% were living with their spouses. While 60% of males never engaged in physical activity, the same was higher in older women (78% never engaged in physical activity). Additionally, 26% of older adults belonged to the SC/ST caste, and almost 70% lived in a rural residence.

**Table 1:** Socioeconomic profile of older adults and percentage of older adults with successful aging by gender in India, 2017–18

Background characteristics	Distribution of older adults (60 + years)				Older adults with successful ageing			
	**Male**		**Female**		**Male**	**Female**	**Difference**^**(a)**^	
	**μ/N**	**SD/Col_%**	**μ/N**	**SD/Col_%**	**Row_%**	**Row_%**	**Row_%**	***p*-value**^**(b)**^
***Individual factors***								
**Mean age (in years)**	69.3	0.1	69.1	0.1				
**Age**								
Young-old	8,730	57.8	9,678	59.1	39.0	29.2	9.8	< 0.001
Old-old	4,702	31.1	4,803	29.4	28.8	20.3	8.5	< 0.001
Oldest-old	1,666	11.0	1,886	11.5	21.5	14.2	7.3	< 0.001
**Education**								
Not educated/primary not completed	8,019	53.1	13,314	81.4	35.0	26.0	9.0	< 0.001
Primary	2,235	14.8	1,297	7.9	34.8	21.0	13.9	< 0.001
Secondary	3,096	20.5	1,297	7.9	33.7	20.0	13.8	< 0.001
Higher	1,748	11.6	458	2.8	27.8	17.5	10.3	< 0.001
**Working status**								
Working	6,613	43.8	3,108	19.0	46.0	37.2	8.8	< 0.001
Never worked/Retired	7,907	52.4	5,593	34.2	24.3	22.0	2.4	< 0.001
Currently not working	578	3.8	7,665	46.8	26.3	22.0	4.3	< 0.001
**Marital status**								
Currently married	12,242	81.1	7,211	44.1	34.2	29.4	4.8	< 0.001
Widowed	2,489	16.5	8,837	54.0	31.6	21.2	10.4	< 0.001
Others	366	2.4	318	2.0	39.4	25.1	14.3	0.013
**Living arrangement**								
Living alone	380	2.5	1,397	8.5	32.9	20.2	12.7	< 0.001
Living with spouse	3,929	26.0	2,485	15.2	28.5	28.7	-0.2	< 0.001
Living with children and spouse	10,205	67.6	11,268	68.9	36.2	25.1	11.1	0.076
Living with others	583	3.9	1,216	7.4	31.6	20.8	10.8	< 0.001
**Tobacco consumption**								
No	6,197	41.1	12,706	77.6	30.4	24.4	6.0	< 0.001
Yes	8,901	59.0	3,660	22.4	36.4	26.5	9.9	< 0.001
**Alcohol consumption**								
No	10,939	72.5	15,943	97.4	33.5	24.6	8.9	< 0.001
Yes	4,159	27.6	423	2.6	35.1	36.4	-1.4	0.437
**Physical activity status**								
Frequent	3,706	24.6	1,966	12.0	44.2	28.5	15.8	< 0.001
Rare	2,360	15.6	1,672	10.2	43.9	33.3	10.6	< 0.001
Never	9,031	59.8	12,729	77.8	27.1	23.2	3.8	< 0.001
***Household factors***								
**MPCE quintile**								
Poorest	3,145	20.8	3,681	22.5	38.3	28.7	9.6	< 0.001
Poorer	3,219	21.3	3,611	22.1	37.0	26.6	10.4	< 0.001
Middle	3,262	21.6	3,331	20.4	34.5	27.1	7.4	< 0.001
Richer	2,902	19.2	3,136	19.2	32.3	22.0	10.3	< 0.001
Richest	2,570	17.0	2,607	15.9	25.8	17.8	7.9	< 0.001
**Religion**								
Hindu	12,386	82.0	13,484	82.4	34.9	25.7	9.1	< 0.001
Muslim	1,769	11.7	1,781	10.9	29.4	17.3	12.1	< 0.001
Christian	388	2.6	511	3.1	32.5	29.5	3.0	0.003
Others	555	3.7	590	3.6	27.9	24.6	3.3	< 0.001
**Caste**								
Scheduled Caste	2,836	18.8	3,113	19.0	37.6	24.1	13.6	< 0.001
Scheduled Tribe	1,166	7.7	1,389	8.5	41.1	40.5	0.6	< 0.001
Other Backward Class	6,925	45.9	7,308	44.7	33.4	24.8	8.7	< 0.001
Others	4,172	27.6	4,556	27.8	30.2	20.9	9.3	< 0.001
**Place of residence**								
Rural	10,879	72.1	11,322	69.2	36.8	27.3	9.4	< 0.001
Urban	4,219	28.0	5,044	30.8	26.6	19.4	7.2	< 0.001
**Region**								
North	1,863	12.3	2,096	12.8	32.1	26.6	5.6	< 0.001
Central	3,395	22.5	3,202	19.6	42.4	31.7	10.7	< 0.001
East	3,713	24.6	3,729	22.8	35.0	28.0	7.1	< 0.001
Northeast	437	2.9	497	3.0	41.6	31.1	10.4	< 0.001
West	2,457	16.3	2,941	18.0	26.8	18.6	8.2	< 0.001
South	3,233	21.4	3,900	23.8	29.1	19.4	9.7	< 0.001
**Total**	**15,098**	**100.0**	**16,366**	**100.0**	**33.9**	**24.9**	**9.0**	**< 0.001**

*μ* Mean, *SD* Standard deviation, *N* Number of older adults, *Col_%* Column percentage, *Row_%* Row percentage of older adults who experienced successful aging

(a) Difference in the percentage of male and female older adults

(b) *p*-values of proportion test of male–female older adults who experienced successful aging

### Estimates from bivariate analysis

Table 1 gives the bivariate distribution of male and female older adults with successful aging by selected explanatory variables. We observed significant gender differences in successful aging by the individual, household and community characteristics. There was significant gender differential in successful aging (male: 34% and female: 25%; difference: 9%; *p*-value: < 0.001). A higher proportion of older men had experienced successful aging across all age groups than their women counterparts. Moreover, among older adults with successful aging, a higher proportion of males had no formal schooling (35%), were working (46%), were living alone (33%), and had frequent physical activity (44%) in comparison to their female counterparts (26, 37, 20 and 29% respectively). Coming to the poorest quintile household, we observed that 38% of older men experienced successful aging compared to 29% among women. Similarly, 37 and 27% of older men and women living in rural areas experienced successful aging. Moreover, these differences by gender were statistically significant at the 1% level.

### Logistic regression estimates of successful aging

Table 2 presents the logistic regression estimates for successful aging among older adults in India. As mentioned earlier, the odds ratio of greater than 1 for a given category of the independent variable denotes higher odds of successful aging, given the effect of all other independent variables remain constant. We found that older women had lower odds [AOR: 0.87; CI: 0.81, 0.94] of successful aging than older men in the study. Moreover, young-old adults had higher odds of successful aging compared to oldest-old adults [AOR: 1.73; CI: 1.57, 1.91]. Working older adults had higher odds of successful aging than older adults who were not working [AOR: 1.66; CI: 1.53, 1.81]. Older adults who were currently married had higher odds of successful aging than widowed older adults [AOR: 1.21; CI: 1.13, 1.30]. Older adults living with their children and spouse had significantly higher odds of successful aging than older adults living with others [AOR: 1.20; CI: 1.05, 1.37]. Older adults who did not consume alcohol had significantly higher odds of successful aging than older adults who consumed alcohol [AOR: 1.09; CI: 1.01, 1.17]. Also, older adults in urban areas had lower odds of successful aging [AOR: 0.92; CI: 0.87, 0.98] than their rural counterparts.

**Table 2:** Logistic regression estimates for successful aging among older adults in India, 2017–18

Background characteristics	AOR (95% CI)
***Individual factors***	
**Gender**	
Male	Ref
Female	0.87*(0.81,0.94)
**Age**	
Young-old	1.73*(1.57,1.91)
Old-old	1.30*(1.18,1.44)
Oldest-old	Ref
**Education**	
Not educated/primary not completed	Ref
Primary	1.00(0.92,1.08)
Secondary	1.03(0.95,1.12)
Higher	0.95(0.85,1.06)
**Working status**	
Working	1.66*(1.53,1.81)
Never worked/Retired	0.93(0.86,1)
Currently not working	Ref
**Marital status**	
Currently married	1.21*(1.13,1.3)
Widowed	Ref
Others	1.3*(1.1,1.54)
**Living arrangement**	
Living alone	1.23*(1.04,1.46)
Living with spouse	1.1(0.95,1.28)
Living with children and spouse	1.2*(1.05,1.37)
Living with others	Ref
**Tobacco consumption**	
No	1(0.94,1.06)
Yes	Ref
**Alcohol consumption**	
No	1.09*(1.01,1.17)
Yes	Ref
**Physical activity status**	
Frequent	Ref
Rare	1.34*(1.25,1.44)
Never	1.29*(1.19,1.39)
***Household factors***	
**MPCE quintile**	
Poorest	Ref
Poorer	0.94(0.87,1.01)
Middle	0.91*(0.84,0.98)
Richer	0.79*(0.73,0.86)
Richest	0.69*(0.63,0.75)
**Religion**	
Hindu	Ref
Muslim	0.78*(0.71,0.85)
Christian	1.06(0.96,1.18)
Others	0.94(0.83,1.07)
**Caste**	
Scheduled Caste	0.99(0.91,1.08)
Scheduled Tribe	1.38*(1.26,1.51)
Other Backward Class	1.05(0.98,1.12)
Others	Ref
**Place of residence**	
Rural	Ref
Urban	0.92*(0.87,0.98)
**Region**	
North	Ref
Central	1.22*(1.11,1.34)
East	0.93(0.85,1.02)
Northeast	1.34*(1.2,1.49)
West	0.69*(0.63,0.77)
South	0.73*(0.67,0.8)
**Analytical sample size**	**31,464**

*AOR* Adjusted odds ratio, *CI* Confidence interval, *Ref* Reference category

^*^ denotes *p*-value < 0.05

Older adults belonging to the richest quintile of household wealth status had lower odds of successful aging in this study [AOR: 0.69, CI: 0.63, 0.75] than their poorest counterparts. Table 3 provides the logistic regression estimates of each component of successful aging with MPCE quintile among older adults in India, stratified by gender. Older adults who belonged to richest quintile were disadvantageous in terms of the components of absence of chronic diseases among males [AOR: 0.49, CI: 0.44, 0.55] and females [AOR: 0.51, CI: 0.45, 0.57], free from depressive symptoms among males [AOR: 0.64, CI: 0.51, 0.81] and free from obesity among females [AOR: 0.50, CI: 0.40, 0.62] compared to the poorer group. Those in the rich category had significantly increased odds in case of high cognitive ability and active social engagements than their poor counterparts.

**Table 3:** Logistic regression estimates for the association between components of successful ageing and MPCE quintile (stratified by sex), LASI 2018–19

Socio-economic indicator	Male, AOR (95% CI)	Female, AOR (95% CI)
**Absence of chronic diseases**		
**MPCE quintile**		
Poorest	Ref	Ref
Poorer	0.8*(0.71,0.89)	0.84*(0.76,0.93)
Middle	0.76*(0.68,0.85)	0.69*(0.63,0.77)
Richer	0.63*(0.56,0.7)	0.59*(0.53,0.66)
Richest	0.49*(0.44,0.55)	0.51*(0.45,0.57)
**Free from disability**		
**MPCE quintile**		
Poorest	Ref	Ref
Poorer	1.06(0.93,1.21)	1.08(0.96,1.21)
Middle	0.93(0.82,1.07)	1.04(0.92,1.17)
Richer	1.06(0.92,1.22)	0.98(0.87,1.1)
Richest	0.96(0.83,1.11)	0.94(0.83,1.06)
**High cognitive ability**		
**MPCE quintile**		
Poorest	Ref	Ref
Poorer	1.11(0.89,1.39)	1.18*(1.02,1.36)
Middle	1.3*(1.03,1.65)	1.35*(1.16,1.57)
Richer	1.37*(1.07,1.75)	1.48*(1.26,1.73)
Richest	1.39*(1.06,1.82)	1.73*(1.46,2.05)
**Free from depressive symptoms**		
**MPCE quintile**		
Poorest	Ref	Ref
Poorer	1.02(0.82,1.27)	1.04(0.87,1.24)
Middle	0.92(0.74,1.15)	1.34*(1.10,1.62)
Richer	0.78*(0.63,0.98)	0.99(0.82,1.2)
Richest	0.64*(0.51,0.81)	0.86(0.71,1.05)
**Active social engagement in life**		
**MPCE quintile**		
Poorest		
Poorer	1.34*(1.12,1.62)	1.33*(1.14,1.56)
Middle	1.65*(1.36,2.01)	1.50*(1.28,1.77)
Richer	1.58*(1.29,1.93)	1.66*(1.40,1.97)
Richest	1.77*(1.42,2.19)	1.53*(1.28,1.83)
**Free from obesity**		
**MPCE quintile**		
Poorest	Ref	Ref
Poorer	1.22(0.85,1.74)	0.87(0.69,1.09)
Middle	0.98(0.7,1.38)	0.87(0.69,1.09)
Richer	0.75(0.54,1.04)	0.56*(0.45,0.69)
Richest	0.77(0.55,1.08)	0.50*(0.40,0.62)

*AOR* Adjusted odds ratio, *CI* Confidence interval, *Ref* Reference category

^*^ denotes *p*-value < 0.05; the estimates were adjusted for individual factors and household factors

### Decomposition of gender differences in successful aging

Table 4 shows the contribution of individual and household characteristics to gender inequality in successful aging among older adults. The results show significant gender inequality in successful aging (Coefficient: 0.090; *p*-value < 0.05). Further, 88% of the gender difference can be explained by the differences in distributions of characteristics (Coef: 0.082; *p*-value < 0.05).

**Table 4:** Multivariate logistic regression decomposition estimates for gender differentials in successful ageing among older adults in India, 2017–18

Characteristics	Due to difference in characteristics			Due to difference in coefficients		
	**Coef**	**SE**	**Percent**	**Coef**	**SE**	**Percent**
***Individual factors***			*35.4*			*-84.86*
**Age**						
Young-old	-0.00168*	0.000	-1.8	-0.00762	0.024	-8.3
Old-old	0.00122*	0.000	1.3	0.00894	0.015	9.7
Oldest-old	-	-	-	-	-	-
**Education**						
Not educated/primary not completed	-	-	-	-	-	-
Primary	0.00017	0.001	0.2	0.00279	0.004	3.0
Secondary	0.00186	0.001	2.0	0.00438	0.006	4.7
Higher	0.00013	0.001	0.1	0.00174	0.003	1.9
**Working status**						
Working	0.02536*	0.004	27.5	0.00253	0.009	2.7
Never worked/Retired	0.00018	0.003	0.2	0.00513	0.016	5.6
Currently not working	-	-	-	-	-	-
**Marital status**						
Currently married	-0.00092	0.004	-1.0	-0.05363	0.061	-58.1
Widowed	-	-	-	-	-	-
Others	0.00003	0.000	0.0	-0.00123	0.002	-1.3
**Living arrangement**						
Living alone	-0.00184	0.001	-2.0	-0.00226	0.006	-2.5
Living with spouse	-0.00121	0.002	-1.3	-0.01371	0.018	-14.9
Living with children and spouse	-0.00001	0.000	0.0	-0.06500	0.083	-70.5
Living with others	-	-	-	-	-	-
**Tobacco consumption**						
No	0.00299	0.002	3.2	-0.02712	0.037	-29.4
Yes	-	-	-	-	-	-
**Alcohol consumption**						
No	-0.00482*	0.002	-5.2	0.04776	0.065	51.8
Yes	-	-	-	-	-	-
**Physical activity status**						
Frequent	0.00813*	0.001	8.8	0.01071	0.013	11.6
Rare	0.00314*	0.000	3.4	0.00829	0.010	9.0
Never						
***Household factors***			*0.6*			*-22.0*
**MPCE quintile**						
Poorest	-	-	-	-	-	-
Poorer	0.00011*	0.000	0.1	-0.00676	0.010	-7.3
Middle	0.00006*	0.000	0.1	-0.00891	0.012	-9.7
Richer	-0.00021*	0.000	-0.2	-0.00819	0.011	-8.9
Richest	-0.00113*	0.000	-1.2	-0.01176	0.015	-12.7
**Religion**						
Hindu	-	-	-	-	-	-
Muslim	-0.00003	0.000	0.0	0.01262	0.015	13.7
Christian	-0.00002	0.000	0.0	-0.00228	0.005	-2.5
Others	-0.00001	0.000	0.0	0.00079	0.003	0.9
**Caste**						
Scheduled Caste	-0.00004	0.000	0.0	0.01144	0.014	12.4
Scheduled Tribe	-0.00034*	0.000	-0.4	-0.00038	0.006	-0.4
Other Backward Class	0.00008	0.000	0.1	0.00087	0.010	0.9
Others	-	-	-	-	-	-
**Place of residence**						
Rural	-	-	-	-	-	-
Urban	0.00053*	0.000	0.6	-0.02035	0.025	-22.1
**Region**						
North	-	-	-	-	-	-
Central	0.00061*	0.000	0.7	0.00419	0.007	4.5
East	-0.00029*	0.000	-0.3	-0.00706	0.010	-7.7
Northeast	-0.00012*	0.000	-0.1	-0.00133	0.005	-1.4
West	0.00073*	0.000	0.8	0.00860	0.011	9.3
South	0.00057*	0.000	0.6	0.00818	0.012	8.9
**Constant**				0.02545	0.079	27.6
**Total**	0.08152*	**0.008**	**88.4**	**0.01074**	**0.009**	**11.6**

*Coef* Coefficient, *SE* Standard error

^*^ denotes *p*-value < 0.05

Considering the differences due to characteristics, we observed that most of the gender gap in successful aging would be reduced (28% reduction in the observed gap) if women had similar status as currently working as their male counterparts. Moreover, bringing the level of frequent physical activity in women to the same levels observed in men would reduce the gender gap by 9%. At the aggregate-level, we found that 35% of gender difference in successful aging is attributable to difference in the distribution of individual among older women and men. Considering the differences due to coefficients, a significant gender gap (around 10%) would have reduced if the similar proportion of older women had at least primary level of education as men. Also, if older men had an equal chance of living in a rural community as their female counterparts, it would facilitate a 22% decrease in the gender gap in successful aging.

## Discussion

This article aimed to explore the frequencies and gender differences in successful aging in older men and women in India and the factors contributing to those differences. Using the definition of Rowe and Kahn, the study identified 34.3% of older men and 25.6% of older women as successfully aging. Multiple studies have used and operationalized various definitions, including a few with a single component of the absence of diseases and found the frequency of successful aging ranging from < 1% to > 90% of the participants [33]. Therefore, comparing the current finding with the existing studies is difficult. Using a similar model of successful aging, a recent multi-country study in China, Korea and Japan found that 17.6% of the population aged between 65 and 75 were successful agers [34]. Similarly, a survey among Chilean older adults using a multidimensional 20-item successful aging inventory devised by Troutman [35] identified more than 64% of the participants as aging successfully [36].

Importantly, our data showed significant gender differences, with older men having higher odds of successful aging than women. This finding was consistent with previous studies, suggesting that women have higher morbidity than men due to acute and chronic physical and psychological disorders, and even when variables related to reproduction were dropped, the variations in morbidity remained [37–40]. Also, the assessment of functional health, including measures of difficulty in executing functions related to ADL, such as eating, getting dressed, washing, and using the bathroom, demonstrated significant disparities in favour of men [41, 42]. Similarly, previous studies show that physical restrictions, such as ADL, affect older women more than men [43, 44]. Again, better cognitive functioning among men than women could be the basis of many other healthier personal choices, allowing men to age more successfully than their female counterparts [45–47]. Additionally, due to higher widowhood rates, social isolation and loneliness among older women are considered the most concerning issue in successful aging studies [48–51].

Several explanations have been documented on the gender variations in morbidity. For example, men are more likely to suffer from diseases such as cancer, hypertension and heart disease [52–54]; women, on the other hand, have higher rates of chronic conditions such as arthritis, osteoporosis, related fractures and depression. These disorders harm mental and physical health but have a lower risk of death than cancer, hypertension, and heart disease [39, 55–57]. On the other hand, women have a higher incidence and prevalence of dementia than males, partly owing to women’s longer life expectancy and the increasing risk of different diseases as they age [58, 59]. Another common reason for the gender variation in morbidity is that men and women have different lifestyles strongly linked to mortality and have been more widespread in men for years [60]. In parallel with this, the present study found that physical activity contributed majorly to the differentials in experiencing successful aging. This could partially be explained by the possible reverse causality that successful agers may have greater physical reserves to undertake physical activity. Although causal connection is not established, older men and women should increase physical activity appropriately by participating in household-related activities, physical exercises, and voluntary activities as physical activity could help preserve health and practical functionality in older people and reduce the risk of chronic diseases [61].

While smoking and drinking are regarded as dangers that primarily influence men’s morbidity and death, the presently evident obesity pandemic is more prevalent among women and poses a particular challenge to their health and functioning [62]. Hence, interventions that address the adverse dietary patterns and malnutrition among older people should be developed through a gender lens. In addition, men might have variables related to healthier lifestyles and better qualitative aspects of social interactions during their earlier lives, giving them a higher chance of aging successfully than women. As evident in past studies, social disadvantage experienced by women in the form of increased responsibilities for housework, lower education, and low socioeconomic status led to worse access to healthcare and higher morbidity [41–43]. In support of this, our findings suggest that marital status and living arrangements significantly contribute to the sex differences in successful aging.

Interestingly, the current results showed an advantage for economically poor (based on household MPCE quintile) and rural-dwelling older adults in successful aging, which is in variance with an extant study showing a lower score of successful aging among socioeconomically poor people and rural residents [63]. This finding could be attributed to the increased likelihood of under-diagnosis and under-reporting of diseases among lower socioeconomic groups in India, as suggested by multiple studies [64–66]. A recent study indicates that more than half of India’s urban–rural gradient in disability is attributable to education and household wealth distribution compared with less than 20% in China [67]. Hence, the successful aging disparity can partially be explained by the differential poverty and illiteracy rates in urban and rural areas. Also, as evidence suggests, older people with higher socioeconomic status and those who reside in urban areas are more likely to have several lifestyle diseases including obesity [22, 68, 69] which may ultimately result in poor mental and physical performance and lower rate of successful aging.

One of the study’s limitations is that we cannot make any causal inferences about gender differences in experiencing different rates of successful aging for older people in India due to the cross-sectional design. Secondly, the definition of successful aging can raise special attention because it is highly multidimensional and heterogeneous [33]. We used the definition according to the existing literature classifying a few individuals as aging successfully which can probably affect reducing or increasing the number of associated variables that are particular in the specific socio-cultural context.

## Conclusion

This study adds to the literature on the interplay between gender and experiencing successful aging. We found that women consistently had a lower score in successful aging, which is attributed to several socioeconomic and behavioural factors including education, household consumption quintile and work status. The findings suggest that within the changing socio-demographic and epidemiological landscape of Indian society, it is essential that public health initiatives be developed with a gender perspective to promote social and mental wellbeing and prevent physical and functional disability, considering that women appear to face more significant disadvantages than men. More studies must be conducted to explore the reasons for such differences and what factors in low-income countries create differences among older men and women in achieving successful aging. The future studies should also investigate the counterintuitive finding related to the lower rate of successful aging among people belonging to urban regions and the rich consumption quintile.

## Data Availability

The data are available at the Gateway to Global Aging Data (www.g2aging.org).

## References

[1] United Nations. World population prospects 2019. Department of Economic and Social Affairs. World Population Prospects 2019. New York. 2019.

[2] Chen LK (2019). Integrated care for older people: solutions to care fragmentation. Aging Med Healthc.

[3] Rowe JW, Kahn RL (1987). Human aging: usual and successful. Science.

[4] Baltes PB, Baltes MM. Psychological perspectives on successful aging: the model of selective optimization with compensation. Successful aging. 1990. Successful aging: Perspectives from the behavioral sciences (pp. 1–34). Cambridge University Press. 10.1017/CBO9780511665684.003.

[5] Rowe JW, Kahn RL (1997). Successful Aging. Cerontologist.

[6] McLaughlin SJ, Connell CM, Heeringa SG, Li LW, Roberts JS (2010). Successful aging in the United States: Prevalence estimates from a national sample of older adults. J Gerontol Ser B Psychol Sci Soc Sci.

[7] Ng TP, Broekman BFP, Niti M, Gwee X, Kua EH (2009). Determinants of successful aging using a multidimensional definition among chinese elderly in singapore. Am J Geriatr Psychiatry.

[8] Hsu HC, Jones BL (2012). Multiple trajectories of successful aging of older and younger cohorts. Gerontologist.

[9] Feng Q, Son J, Zeng Y (2015). Prevalence and correlates of successful ageing: a comparative study between China and South Korea. Eur J Ageing.

[10] Idler EL (2003). Discussion: Gender differences in self-rated health, in mortality, and in the relationship between the two. Gerontologist.

[11] Oksuzyan A, Brønnum-Hansen H, Jeune B (2010). Gender gap in health expectancy. Eur J Ageing.

[12] Kaneda T, Zimmer Z, Xianghua F, Zhe T (2009). Gender differences in functional health and mortality among the Chinese elderly: testing an exposure versus vulnerability hypothesis. Res Aging.

[13] Carmel S, Bernstein JH (2003). Gender differences in physical health and psychosocial well being among four age-groups of elderly people in Israel. Int J Aging Hum Dev.

[14] Stevenson B, Wolfers J (2009). The paradox of declining female happiness. Am Econ J Econ Policy.

[15] Bongaarts J, Zimmer Z (2002). Living arrangements of older adults in the developing world: an analysis of demographic and health survey household surveys. J Gerontol B Psychol Sci Soc Sci.

[16] Yang K, Victor CR (2008). The prevalence of and risk factors for loneliness among older people in China. Ageing Soc.

[17] Marquand R. Love and money reshape family in China. Christ Sci Monit. 2004;1(1).

[18] Lee WJ, Peng LN, Lin MH, Loh CH, Chen LK (2020). Determinants and indicators of successful ageing associated with mortality: a 4-year population-based study. Aging.

[19] International Institute for Population Sciences (IIPS), NPHCE, MoHFW, Harvard T. H. Chan School of Public Health (HSPH), The University of Southern California (USC). Longitudinal Ageing Study in India (LASI) Wave 1. India Rep. Mumbai, India; 2020.

[20] Luo H, Ren X, Li J, Wu K, Wang Y, Chen Q (2020). Association between obesity status and successful aging among older people in China: evidence from CHARLS. BMC Public Health.

[21] Rowe J, Kahn R (2000). Successful aging and disease prevention. Adv Ren Replace Ther.

[22] Srivastava S, Joseph KJV, Dristhi D, Muhammad T (2021). Interaction of physical activity on the association of obesity-related measures with multimorbidity among older adults: a population-based cross-sectional study in India. BMJ Open.

[23] Srivastava S, Muhammad T (2020). Violence and associated health outcomes among older adults in India: a gendered perspective. SSM Popul Health.

[24] Muhammad T, Srivastava S. Why Rotational Living Is Bad for Older Adults ? Evidence from a Cross- Sectional Study in India. J Popul Ageing. 2020;1. 10.1007/s12062-020-09312-4

[25] Sharma P, Maurya P, Muhammad T (2021). Number of chronic conditions and associated functional limitations among older adults: cross-sectional findings from the longitudinal aging study in India. BMC Geriatr.

[26] Pandav R, Fillenbaum G, Ratcliff G, Dodge H, Ganguli M (2002). Sensitivity and specificity of cognitive and functional screening instruments for dementia: the Indo-U.S. dementia epidemiology study. J Am Geriatr Soc.

[27] Muhammad T, Meher T, Sekher TV (2021). Association of elder abuse, crime victimhood and perceived neighbourhood safety with major depression among older adults in India: a cross-sectional study using data from the LASI baseline survey (2017–2018). BMJ Open.

[28] Zhang J, Xu L, Li J, Sun L, Qin W (2020). Association between obesity-related anthropometric indices and multimorbidity among older adults in Shandong, China: a cross-sectional study. BMJ Open.

[29] Fan C, Wang L, Wei L (2017). Comparing two tests for two rates. Am Stat.

[30] Osborne J, King JE. Binary logistic regression. Best practices in quantitative methods. SAGE Publications, Inc.; 2011. pp. 358–384. 10.4135/9781412995627.d29

[31] Powers DA, Yoshioka H, Yun MS (2011). Mvdcmp: multivariate decomposition for nonlinear response models. Stata J.

[32] Tiruneh SA, Lakew AM, Yigizaw ST, Sisay MM, Tessema ZT (2020). Trends and determinants of home delivery in Ethiopia: further multivariate decomposition analysis of 2005–2016 Ethiopian Demographic Health Surveys. BMJ Open.

[33] Cosco TD, Prina AM, Perales J, Stephan BCM, Brayne C (2014). Operational definitions of successful aging: a systematic review. Int Psychogeriatr.

[34] Nakagawa T, Cho J, Yeung DY (2021). Successful Aging in East Asia: comparison among China, Korea, and Japan. J Gerontol Ser B.

[35] Troutman M, Nies MA, Small S, Bates A (2011). The development and testing of an instrument to measure successful aging. Res Gerontol Nurs.

[36] Gallardo-Peralta LP, Mayorga Muñoz C, Soto HA (2020). Health, social support, resilience and successful ageing among older Chilean adults. Int Soc Work.

[37] Boerma T, Hosseinpoor AR, Verdes E, Chatterji S (2016). A global assessment of the gender gap in self-reported health with survey data from 59 countries. BMC Public Health.

[38] Nathanson CA (1975). Illness and the feminine role: a theoretical review. Soc Sci Med.

[39] Waldron I (1983). Sex differences in illness incidence, prognosis and mortality: Issues and evidence. Soc Sci Med.

[40] Verbrugge LM (1985). Gender and health: an update on hypotheses and evidence. J Health Soc Behav.

[41] Alexandre Tda S, Corona LP, Nunes DP, Santos JLF, Duarte YA de O, Lebrão ML (2012). Gender differences in incidence and determinants of disability in activities of daily living among elderly individuals: SABE study. Arch Gerontol Geriatr.

[42] Al Hazzouri AZ, Sibai AM, Chaaya M, Mahfoud Z, Yount KM (2011). Gender differences in physical disability among older adults in underprivileged communities in Lebanon. J Aging Health.

[43] Zunzunegui MV, Alvarado BE, Guerra R, Gómez JF, Ylli A, Guralnik JM (2015). The mobility gap between older men and women: the embodiment of gender. Arch Gerontol Geriatr.

[44] Rotarou ES, Sakellariou D (2019). Structural disadvantage and (un)successful ageing: gender differences in activities of daily living for older people in Chile. Crit Public Health.

[45] Lee Y, Shinkai S (2005). Correlates of cognitive impairment and depressive symptoms among older adults in Korea and Japan. Int J Geriatr Psychiatry.

[46] Muhammad T, Srivastava S, Sekher TV (2021). Association of self-perceived income sufficiency with cognitive impairment among older adults: a population-based study in India. BMC Psychiatry.

[47] Muhammad T, Govindu M, Srivastava S (2021). Relationship between chewing tobacco, smoking, consuming alcohol and cognitive impairment among older adults in India: a cross-sectional study. BMC Geriatr.

[48] Cohen-Mansfield J, Parpura-Gill A (2007). Loneliness in older persons: A theoretical model and empirical findings. Int Psychogeriatr.

[49] Hawkley LC, Burleson MH, Berntson GG, Cacioppo JT (2003). Loneliness in everyday life: cardiovascular activity, psychosocial context, and health behaviors. J Pers Soc Psychol.

[50] Prince MJ, Harwood RH, Blizard RA, Thomas A, Mann AH (1997). Social support deficits, loneliness and life events as risk factors for depression in old age. The Gospel Oak Project VI. Psychol Med.

[51] Sorkin D, Rook KS, Lu JL (2002). Loneliness, lack of emotional support, lack of companionship, and the likelihood of having a heart condition in an elderly sample. Ann Behav Med.

[52] Xu X, Mishra GD, Dobson AJ, Jones M (2018). Progression of diabetes, heart disease, and stroke multimorbidity in middle-aged women: a 20-year cohort study. PLoS Med.

[53] Beaglehole R, Epping-Jordan JA, Patel V, Chopra M, Ebrahim S, Kidd M (2008). Improving the prevention and management of chronic disease in low-income and middle-income countries: a priority for primary health care. The Lancet.

[54] Teh JKL, Tey NP, Ng ST (2014). Ethnic and gender differentials in non-communicable diseases and self-rated health in Malaysia. PLoS ONE.

[55] Dhak B, Mutharayappa R. Gender differential in disease burden: its role to explain gender differential in mortality. ISEC Work Pap Ser. Bangalore, India. 221. 2009; 1–17.

[56] Joshi R, Cardona M, Iyengar S, Sukumar A, Raju CR, Raju KR (2006). Chronic diseases now a leading cause of death in rural India - mortality data from the Andhra Pradesh Rural Health Initiative. Int J Epidemiol.

[57] Chireh B, D’Arcy C (2018). Pain and self-rated health among middle-aged and older Canadians: an analysis of the Canadian community health survey - healthy aging. BMC Public Health.

[58] Brown GC (2015). Living too long: The current focus of medical research on increasing the quantity, rather than the quality, of life is damaging our health and harming the economy. EMBO Rep.

[59] Perna L, Wahl HW, Mons U, Saum KU, Holleczek B, Brenner H (2015). Cognitive impairment, all-cause and cause-specific mortality among non-demented older adults. Age Ageing.

[60] McCartney G, Mahmood L, Leyland AH, David Batty G, Hunt K (2011). Contribution of smoking-related and alcohol-related deaths to the gender gap in mortality: evidence from 30 European countries. Tob Control.

[61] Haskell WL, Lee IM, Pate RR, Powell KE, Blair SN, Franklin BA (2007). Physical activity and public health: Updated recommendation for adults from the American College of Sports Medicine and the American Heart Association. Med Sci Sports Exerc.

[62] Whitson HE, Landerman LR, Newman AB, Fried LP, Pieper CF, Cohen HJ (2010). Chronic medical conditions and the sex-based disparity in disability: the cardiovascular health study. J Gerontol Ser Biol Sci Med Sci.

[63] Ding W, Zhang Y, Zhang L, Wang Z, Yu J, Ji H (2020). Successful aging and environmental factors in older individuals in urban and rural areas: a cross-sectional study. Arch Gerontol Geriatr.

[64] Vellakkal S, Subramanian SV, Millett C, Basu S, Stuckler D, Ebrahim S (2013). Socioeconomic inequalities in non-communicable diseases prevalence in India: disparities between self-reported diagnoses and standardized measures. PLoS ONE.

[65] Basu S, King AC (2013). Disability and chronic disease among older adults in India: detecting vulnerable populations through the WHO SAGE study. Am J Epidemiol.

[66] Arokiasamy P, Uttamacharya, Kowal P, Capistrant BD, Gildner TE, Thiele E, et al. Chronic noncommunicable diseases in 6 low- and middle-income countries: Findings from wave 1 of the world health organization’s Study on Global Ageing and Adult Health (SAGE). Am J Epidemiol. 2017;185:414–428. 10.1093/aje/kww125 10.1093/aje/kww125 PMC6075549 28399566

[67] Stewart Williams J, Norström F, Ng N (2017). Disability and ageing in China and India - Decomposing the effects of gender and residence. Results from the WHO study on global AGEing and adult health (SAGE). BMC Geriatr.

[68] Muhammad T, Boro B, Kumar M, Srivastava S (2022). Gender differences in the association of obesity-related measures with multi-morbidity among older adults in India: evidence from LASI, Wave-1. BMC Geriatr.

[69] Nethan S, Sinha D, Mehrotra R (2017). Non communicable disease risk factors and their trends in India. Asian Pac J Cancer Prev APJCP.

